# The social implications of using drones for biodiversity conservation

**DOI:** 10.1007/s13280-015-0714-0

**Published:** 2015-10-27

**Authors:** Chris Sandbrook

**Affiliations:** United Nations Environment Programme World Conservation Monitoring Centre, 219 Huntingdon Road, Cambridge, CB3 0DL UK

**Keywords:** Biodiversity conservation, Drones, UAVs, Social impacts, Ethics, Political ecology

## Abstract

Unmanned aerial vehicles, or ‘drones’, appear to offer a flexible, accurate and affordable solution to some of the technical challenges of nature conservation monitoring and law enforcement. However, little attention has been given to their possible social impacts. In this paper, I review the possible social impacts of using drones for conservation, including on safety, privacy, psychological wellbeing, data security and the wider understanding of conservation problems. I argue that negative social impacts are probable under some circumstances and should be of concern for conservation for two reasons: (1) because conservation should follow good ethical practice; and (2) because negative social impacts could undermine conservation effectiveness in the long term. The paper concludes with a call for empirical research to establish whether the identified social risks of drones occur in reality and how they could be mitigated, and for self-regulation of drone use by the conservation sector to ensure good ethical practice and minimise the risk of unintended consequences.

## Introduction

Unmanned aerial vehicles, or ‘drones’, are rapidly gaining in popularity as nature conservation tools. They appear to offer a flexible, accurate and affordable solution to technical challenges of conservation monitoring and law enforcement (Koh and Wich 2012; Anderson and Gaston 2013). However, little attention has been given to the possible social impacts of using drones for conservation (Humle et al. 2014), despite the fact that such issues have been reported for other civil applications of drones (Finn and Wright 2012). This paper reviews these possible impacts, considers their implications for conservation effectiveness, and calls for a programme of research and self-regulation to better understand and mitigate possible risks.

### What are drones?

Drones are self-propelled airborne devices that have no on-board pilot. They are known by various names, including unmanned aerial vehicles (UAVs), unmanned aerial/aircraft systems (UASs; to include ground-based elements to the system) and remotely piloted aircraft systems (RPAS). Some authors make a distinction between devices capable of autonomous flight, which they call drones, and devices controlled by a ground-based operator, which they call remotely piloted vehicles (RPVs; e.g. Finn and Wright 2012), whereas most authors use the word drone in both cases (e.g. Schiffman 2014). Some authors consider the term drone to apply only to military devices (e.g. Mulero-Pazmany et al. 2014), whereas others selectively avoid using the word drone because “negative connotations [associated with military applications] may undermine cooperation with communities” (Paneque-Galvez et al. 2014, p. 1501). In this article, I use the word drone because it is more widely used and understood than technical terms such as UAV, and because it avoids the gendered term ‘unmanned’.

**Fig. 1:**
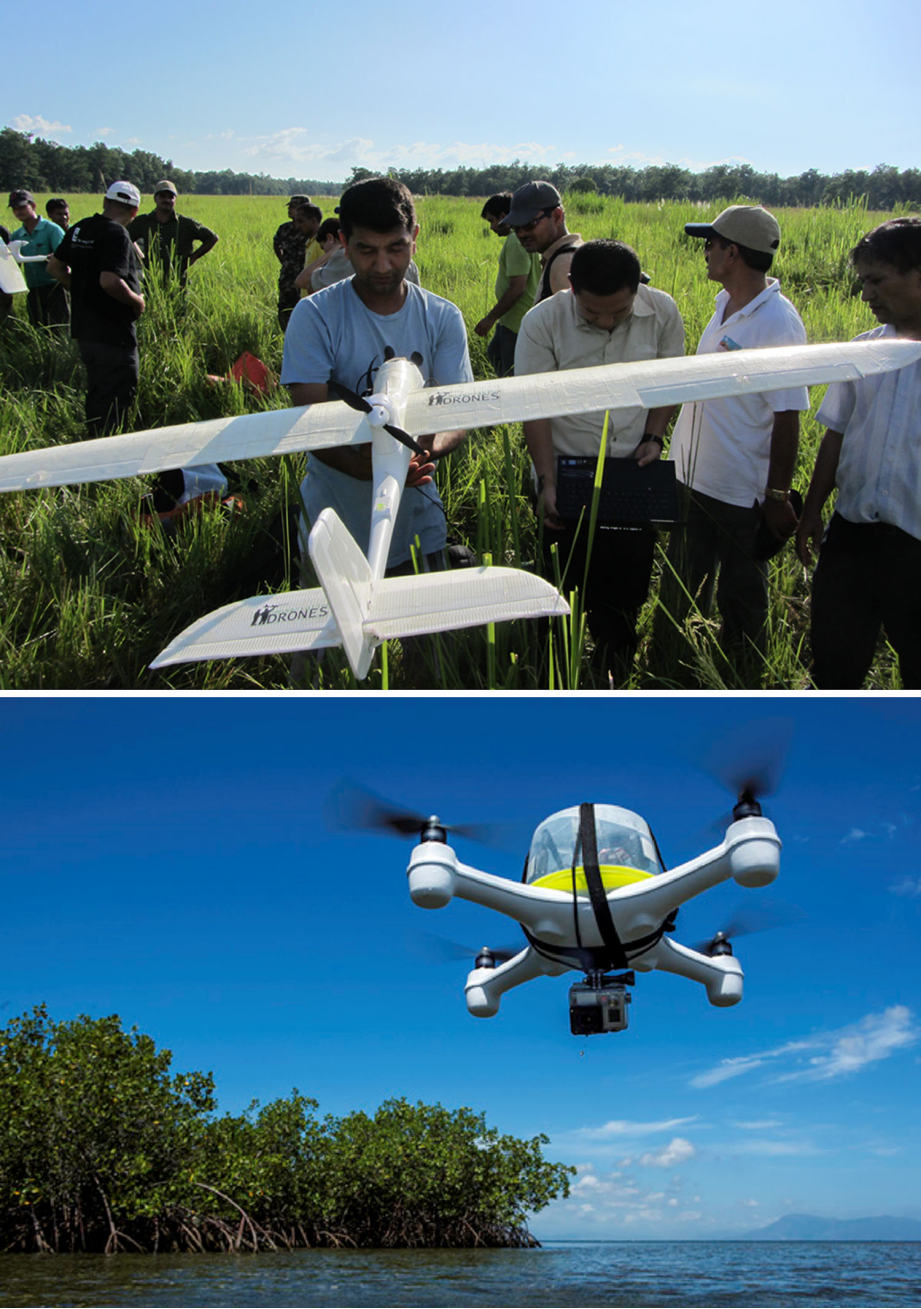
Examples of the application of drones for nature conservation. *Top*: a demonstration by WWF staff of how to launch a fixed wing drone for use in anti-poaching efforts in Nepal. This device was built by Conservation Drones. Photo by Juanita Choo. Photo downloaded from http://conservationdrones.org/2012/09/12/training-of-nepali-park-protection-personnel-on-use-of-conservation-drones-to-stop-wildlife-crime/. *Bottom* a quadcopter rotary wing drone developed by Steve Schill of The Nature Conservancy and his student Jordan Mitchell for mapping marine habitats. This device is capable of take-off and landing on water. Photo by Tim Calver. Photo downloaded from http://blog.nature.org/science/2014/08/11/innovation-drone-mapping-of-coral-reefs-and-the-coastal-zone/

Drones were first developed for military applications around the time of the Second World War (Finn and Wright 2012). In recent years, there has been a great increase in their use, made possible by the miniaturisation and reduction in price of sensory devices like cameras and GPS, largely driven by the smartphone industry (Anderson 2012). Contemporary drones are remarkably diverse (Fig. 1). There are various ways to classify them, including by size, range, endurance and what they carry (Paneque-Galvez et al. 2014). The simplest classification distinguishes between fixed wing and rotary wing devices (although some newer models have both fixed and rotary wings). Fixed wing drones tend to be larger, capable of covering longer distances and carrying heavier loads. The largest fixed wing devices can cost many millions of dollars, are larger than many aircraft with on-board pilots, and can fly for thousands of hours at altitudes above 20 000 m (Watts et al. 2012). Smaller versions, such as the Conservation Drone, are lightweight (~650 g unloaded) and cost less than US $100 for the basic unit (Koh and Wich 2012), although on-board sensory devices tend to increase the total price of the system considerably. Smaller fixed wing drones can often be launched by hand. Fixed wing devices require a linear landing strip, unless they are fitted with a landing parachute. Due to their considerable range, many fixed wing devices can operate beyond line of sight, either autonomously or by remote piloting from the ground (although in many jurisdictions this is not legal). The latter case includes military drones that are flown on combat missions by ‘pilots’ in ground control stations thousands of miles away (Finn and Wright 2012).

Rotary wing drones tend to be smaller than fixed wing devices, and have a reduced range and capacity to carry cargo. However, they are capable of hovering flight and of vertical take-off and landing. This allows them to be deployed in difficult conditions such as under the forest canopy or within caves (e.g. Luo et al. 2014), which would not be possible with a fixed wing device. Rotary wing drones have a high power demand for their wings because they cannot glide, meaning that they can usually only be flown for short distances and for less than 1 h (Watts et al. 2012). Rotary wing drones can be divided into helicopters with a single wing and ‘multicopters’ with multiple wings, (Paneque-Galvez et al. 2014). Most rotary wing devices require line of sight operation by a ground operator (Watts et al. 2012).

## What can drones do?

The capabilities of drones depend on what that they are able to carry. Equipment commonly mounted on drones includes still and video cameras (further subdivided into passively reflected thermal and infrared radiation and emitted thermal radiation sensing devices), audio monitoring devices, loudspeakers, liquid sprayers (e.g. for herbicides), accelerometers, GPS and light emitting devices. In practice, the capabilities of drones are limited mainly by the weight and power demands of their cargo—whilst large drones can carry heavy equipment, smaller units are capable of carrying only very light devices. For example, the Aeryon Scout tricopter drone can carry a 300-g load up to 330 m altitude within a range of 3 km (Watts et al. 2012). A more detailed analysis of drone devices and capabilities is beyond the scope of this paper (see Watts et al. 2012 for a review).

During the twentieth century drones were almost exclusively used for military applications, peaking in the 1990s after the first Gulf War (Nonami 2007). By contrast, the twenty-first century has seen the rapid uptake of drones for civil applications, and it has even been said that “we are entering the drone age” (Anderson 2012). Drones have been used: by police forces around the world for law enforcement tasks such as monitoring crowds, following suspects at night and patrolling international borders (Finn and Wright 2012, and references therein); for precision agriculture (Lelong et al. 2008; Hunt et al. 2010); for fire monitoring and management (Merino et al. 2012); for the delivery of medicines (Hickey 2014); and for the delivery of commercial products and parcels (Arthur 2014; Domino’s Pizza 2014).

## Regulations governing the use of drones

In many parts of the world, the use of drones (for any purpose) is regulated by law, meaning that what drones can do in practice is often limited by regulations rather than their technical capabilities. For example, in the UK, the Civil Aviation Authority allows drones to be flown without a pilot’s licence only if “they weigh less than seven kilograms, stay below 122 metres and within visual line of sight, and are flown away from populated areas and airports” (Anderson 2012). Data collected in the UK through overt monitoring (as with CCTV) must be available on request to those being filmed, whereas covert systems must first be granted permission under the Regulation of Investigatory Powers Act 2000 (Finn and Wright 2012). In the US, small drones face similar regulations to the UK (Watts et al. 2010). A Certificate of Authorisation is required for the use of large drones, which is expensive and difficult to get (Rango and Laliberte 2010), and the use of thermal image cameras to film people is illegal without a warrant under the 4^th^ Amendment (Finn and Wright 2012). Writing about the US, Hardin and Jensen (2011, p. 107) go so far as to say that “the regulations that control small-scale aircraft flight form the greatest obstacle to the technology’s widespread adoption for environmental remote sensing”. Recently, the US National Park Service has taken steps to ban private drones from all US National Parks to minimise safety risks, impacts on wildlife behaviour and harassment of visitors (Guardian 2014). The US state of Illinois is considering banning the use of drones to observe and monitor hunters and anglers (Clemons 2013).

In other parts of the world, South Africa has published draft regulations on the use of drones, but by early 2014, these had not been ratified (Mulero-Pazmany et al. 2014). Kenya has attracted considerable media attention recently for its apparent decision to ban drones (Kariuki 2014), particularly given earlier positive publicity for their use in the same country (Njeru 2014). In some cases, the use of drones may be regulated *de facto* even where *de jure* they have not been banned. For example in Mozambique, the Limpopo National Park was ready to deploy a drone but was blocked by the military, who feared “engines of espionage that might be responding to strange interests” (M. Couto, pers. comm.). Likewise in India, the Ministry of Environment’s plans to use drones at Kaziranga National Park were delayed by the Ministry of Defence (Naveen 2014).

## Conservation applications of drones

The last decade has seen the gradual adoption of drones for use in conservation. Drones used for conservation purposes have included both fixed and rotary wing devices, and have tended to be fairly small, weighing less than 10 kg in total. Applications to date can be broadly divided into two categories: research applications and direct conservation applications. Within the research category, drones have been widely used for the counting and monitoring of wildlife and other biological features that provide data potentially of value for conservation (Koh and Wich 2012; Martin et al. 2012; Marris 2013), going back as far as the 1980s (Tomlins and Lee 1983). Examples include using drones: to measure forest biodiversity (Getzin et al. 2012); to count Dugongs in Australia (Hodgson et al. 2013); to count birds in a range of contexts (Jones et al. 2006; Rodríguez et al. 2012; Sarda-Palomera et al. 2012); and to count elephants in Burkina Faso (Vermeulen et al. 2013). Drones have been used in many different habitats, including the ocean (Lomax et al. 2005), freshwater aquatic habitats (Husson et al. 2014) and rivers (Lejot et al. 2007; Lin et al. 2012). Drones have been promoted for ecological research because of their claimed affordability, flexibility and safety (Anderson and Gaston 2013), although there may be hidden operational costs in practice.

Drones can also be used for more direct conservation applications. One innovative idea is to use them to deliver seeds as part of forest restoration projects (Krupnick 2013; Sutherland et al. 2013). However, the most commonly identified direct application of drones is for law enforcement and the monitoring of illegal activities, particularly in the context of illegal hunting of wildlife (Schiffman 2014). The same characteristics that make drones suitable for ecological research can also be advantageous for boundary patrols (Mulero-Pazmany et al. 2014) and for collecting evidence of illegal activities such as deforestation (Koh and Wich 2012). Drones can be used to catch perpetrators of conservation offences by helping ground-based law enforcement staff to locate and apprehend them (Mulero-Pazmany et al. 2014); to provide high-quality photographic evidence that can be used to secure prosecutions (Snitch 2014) and as a deterrent (Mulero-Pazmany et al. 2014; Schiffman 2014). Drones are considered to be particularly useful when monitoring large areas that are very difficult to cover from the ground (Steiner 2014), especially when used in combination with modelling approaches to predict spatial and temporal patterns of illegal activities (Schiffman 2014; Snitch 2014). Unfortunately, many of the characteristics that make drones useful tools against illegal hunting might also make them very useful to hunters, who could use them in the future to identify and possibly even to dart or kill target animals from a distance.

Despite the wide range of potentially relevant applications, drone use in the conservation sector to date has been largely experimental and dominated by research applications. Direct conservation applications are still in what might be called a pilot or ‘hobby’ phase. However, it seems highly probable that the use of drones for conservation will increase rapidly in the next few years. They are getting more reliable and capable of doing more things all the time, and there are several examples of conservation organisations beginning to actively engage in thinking about how to use them (Vidal 2013; Gorman 2014; Weaver 2014; Wilkie and Rose 2014; WWF 2014a, b).

## The social impacts of drones

Whilst the potential of conservation drones to address technical challenges around data collection and law enforcement is clear, this in itself should not be sufficient justification for their widespread adoption. First, it is necessary to consider whether they may have any other impacts that might affect their suitability and effectiveness. In particular, this article focuses on the potential social impacts of conservation drones—both positive and negative—and how these might relate to their effectiveness as conservation tools. In this section, I consider several such social issues in turn.

### Safety

Drones are generally considered to be safer for the user than piloted aircraft, as there is no pilot to be injured in a crash (Jones et al. 2006; Rango et al. 2006). They may also be safer for people on the ground in a crash scenario because they are usually smaller and therefore likely to do less damage on crashing than larger piloted aircraft (Jones et al. 2006). Many drones feature safety devices to allow them to abandon a pre-planned mission and return to a landing point directly if they experience any problems. On the other hand, being pilotless, drones can be more vulnerable to crashing than piloted aircraft (Finn and Wright 2012; Lee et al. 2013), and accidents might injure people on the ground. Rotary wing devices may be more dangerous than fixed wing devices in cases of engine failure as they tend to fall vertically, whereas fixed wing devices can glide to the ground. It is not yet clear whether the potential advantages of drones outweigh the disadvantages in terms of their safety of operation.

Drones may have an indirect positive impact on the safety of local people on the ground if their use reduces the likelihood of criminals or military forces operating in the area, because they wish to avoid detection.

### Privacy

The use of drones for military and civil applications has attracted a lot of attention to their ethical implications and the ways in which they might infringe privacy and civil liberties (e.g. Sparrow 2009; Kreps and Kaag 2012). In the field of civil applications, drones have been described as a technology of “new surveillance”, alongside closed circuit television and DNA techniques (Marx 1998, 2004). Questions have been raised about whether it is ethically acceptable to monitor people from the air without their knowledge, and at what point this might become an unacceptable infringement of privacy or other human rights, such as freedom of association (see Finn and Wright 2012 for a detailed review). A particular concern with drones is that they can now be small and subtle enough (Luo et al. 2014) to get into spaces that might otherwise be thought of as private. Privacy issues with surveillance technology are not limited to drones—they can also be relevant with high-resolution satellite imagery or traditional aerial photography. In a survey of UK and Australian farmers about their attitudes to being monitored for compliance with legislation using satellite imagery, most farmers were happy to be monitored this way in principle, but 58 % of Australian respondents and 75 % of UK respondents agreed that satellite monitoring was “an invasion of their privacy” (Purdy 2011, p. 205).

Drones used for conservation are very likely to collect information about people that could be used to identify them and what they are doing—indeed in the case of law enforcement applications, this is the deliberate intention. For example, Snitch (2014) advocates using drones to collect vehicle licence plate numbers on public roads outside protected areas in case any are subsequently involved in illegal activities, and Mulero-Pazmany et al. (2014) provide a detailed description of how to use drones for covert observations of potential illegal hunters. Such practices are ethically questionable when taking place on public land, particularly if they target certain groups over others, and are likely to be illegal under existing regulations in many countries. Other technologies have encountered public outcry about invasions of privacy when publishing high-resolution images that were taken without permission, most noticeably in the case of Google StreetView which features photograph quality images of houses and passers-by on urban streets around the World (Strachan 2010).

Drones also have privacy and other ethical implications when used for research. If people or their practices are identifiable from research data, should the people involved be asked for their consent to be surveyed from the air? What might be done with the data, and could there be negative repercussions for the people involved? For example, if data on farming practices collected by a drone reveal illegal forest clearance, will the data be passed on to a law enforcement agency, and might that result in harm to the person on the ground? Such questions are standard in the ethical protocols used by universities and other research agencies for research activities involving interviews or participant observation, which aim to ensure that research does no harm to respondents (e.g. AAG 2009).

### Psychological wellbeing

It has been argued that the use of new technology can be empowering for local groups if it provides them with the means to collect their own data, enforce rules and challenge the claims of others who may wish to mislead them (Lewis and Nkuintchua 2012). Drones could provide these benefits for local people if they were available to be used for community-based forest monitoring to provide carbon measurements and various other useful data (Paneque-Galvez et al. 2014). In this sense, drones in the hands of local people could be socially empowering (Paneque-Galvez et al. 2014).

On the other hand, drones have the potential to cause considerable fear, confusion and hostility among those on the ground. In some cases, this might happen as an accidental consequence of drones being introduced. If people on the ground do not understand, or refuse to believe, why drones are being introduced, they may generate conspiracy theories, suspicions and fantasies, particularly when they are used in remote areas in developing countries which have little prior exposure to electronic devices. Conservation drones have been tested in Tanzania in an area with a prevalent local belief in a supernatural creature called Popo Bawa (Bat Wing in English) which flies at night, paralyses its victim, then swoops down and rapes them. As recently as 2007, there were widespread concerns about this creature (K. Steiner, pers. comm.). It is easy to imagine how the introduction of drones in such a context might trigger a fresh wave of alarm. Similar problems have beset the vaccination campaign for polio, which some believe is a plot to sterilise Nigerian Muslims (Otieno 2013). Likewise, people may recognise a drone for what it is, but have misconceptions about its purpose, perhaps believing it to be sent by a private company, the military, a terrorist group or any number of others. Such perceptions could fuel existing conflicts or create new ones.

The likelihood that drones cause fear or alarm among those on the ground may be influenced by their material characteristics (R. Lamprey, pers comm.). Fixed wing devices are often very quiet and fly at several hundred metres altitude, meaning that they may not be noticed from the ground at all (although this might have ethical consequences, as discussed above). They are also superficially similar in appearance to traditional piloted fixed wing aircraft. By contrast, rotary wing devices tend to be noisy, fly at low altitude and look nothing like previous aircraft, apart from a superficial similarity to helicopters. It seems likely that fixed wing drones will be less obtrusive and more easily acceptable to those on the ground, whereas rotary wing drones will be noticed and might cause alarm to those not expecting to see them. Drones might also negatively affect relations with local people where they replace face-to-face interactions with conservation workers. For example, a ranger on patrol can have a friendly conversation with a passerby, whereas this will not be possible if the patrol is carried out by a drone. Drones may also carry out different conservation actions than would have been the case if the operator were physically present on the ground because of the psychological effect of ‘distancing’ (Sparrow 2009).

Even if people on the ground do know that drones are being deployed for conservation purposes, they may nonetheless feel aggrieved. As Paneque-Galvez et al. (2014, p. 1495) write: “The misuse of drone technology for surveillance without acceptable transparency and communally-agreed rules of engagement could provoke severe conflicts amongst community members (e.g. accusations of privacy violations and spying). Partner organisations could be ultimately blamed for whatever problems that might arise amongst community members as a result of the introduction of drone technology”. Conservation organisations and agencies have a long history of conflict and disputes with local people, derived from evictions, exclusion, limitation of activities and other actions that are contrary to local interests (West et al. 2006). It seems more likely that the use of drones for conservation purposes will be negatively received in places with a history of such conflict.

In some cases, drones might be used deliberately to create fear of punishment as a deterrent against illegal activities. This use of drones is particularly questionable from an ethical standpoint. Writing about efforts to prevent illegal hunting in South Africa, Mulero-Pazmany et al. (2014, p. 9) suggest “performing demonstrations (of drones) to the local communities and appearing in media with awareness campaigns, which could make (local communities) afraid and aware that they can be detected even without notice”. Similarly, Snitch (2014) writes that “the poachers are terrified to go into Balule because the word on the street is that there are machines in the sky that can see at night, and the rangers know where they will walk. This might be voodoo, but it works.” These authors seem to advocate the creation of widespread fear among the local population, using what Michel Foucault called a ‘disciplinary governmentality’ approach that encourages members of the public to internalise norms of pro-conservation behaviour through the belief that they are being watched at all times (Foucault 1977; Fletcher 2010). This approach might be effective in its direct objective of deterring hunters, but the use of fear as a tool of conservation raises obvious ethical questions.

### Data security

Some people may be concerned about how data collected by conservation drones are used and to what ends. It might be acceptable for data to be collected by a law enforcement agency to prevent illegal hunting, but does this still apply if those same data are then sold on to a commercial entity such as an advertiser? Data may also be shared with wider networks such as state security agencies, which have been under attack from those concerned about civil liberties following recent revelations about their activities (e.g. Guardian 2013). Finally, hackers might steal data from drones, which are considered particularly susceptible to this problem as they can be shot down, collected and dismantled by those wishing to get access to data (Hartmann and Steup 2013).

### Wider understanding of conservation problems

The use of drones could help to connect the wider public to issues affecting locations of conservation concern by providing access to up to date high-resolution images and other data. However, there seems a risk that the use of drones may support simplistic and counterproductive narratives about conservation that are prevalent among the general public in cities and countries distant from their use. For example, a recent editorial in the Guardian newspaper (2014) entitled ‘In Praise of Drones’ drew the following comment: “Drones should be sent to Africa to survey and protect the Rhinos. Ideally they should also have hellfire missiles to deal summarily with poachers.” (ArnaudAmalric). This comment reveals the strength of association between drones and military applications that exist in the minds of many people—an association that some users of conservation drones actively promote (e.g. IAPF 2014). It is well-established that conservation problems such as the illegal wildlife trade are highly complex, and the use of drones to address illegal hunting may in itself serve to promote simplistic narratives of ‘good’ conservationists and ‘evil’ poachers, thereby undermining understanding of this complex issue among the wider public (Humle et al. 2014).

## Implications for conservation effectiveness

The use of drones for conservation is in its infancy. There is currently limited evidence regarding their effectiveness as a conservation tool, although early reports have been positive (e.g. Schiffman 2014). For example, writing about the use of drones in South Africa, Snitch (2014) says “Is it working? Yes. There have been no poachings in Balule in the past 8 months. I believe this is largely due to deterrence.” In addition, other remote sensing platforms have proven of great value for conservation (Pettorelli et al. 2014; Van der Wal et al. 2015; Robinson Wilmott et al. 2015), making it likely that drones could also be useful. If drones are positively perceived by people on the ground, they may conceivably generate support for conservation, making conservation actions easier and more effective. This could be an important benefit for conservation, but no data exist at present to suggest its possible scale. Success for conservation from drones could in turn provide new social benefits, as local people might benefit from the ongoing flow of ecosystem services such as ecotourism incomes or pollination of crops, and people living further away might also benefit from services such as climate change mitigation or from the existence value they gain from knowing that rare species are being protected (MA 2005; Saito et al. 2015).

On the other hand, a number of negative impacts of drones for conservation effectiveness seem plausible. First, data collected by drones might fall into the hands of the wrong people. This could happen because of hacking, or because corrupt officials sell the data or use them themselves for personal gain. Such data might actually facilitate illegal activity by providing information on wildlife locations and law enforcement efforts to criminals. Second, several of the possible social impacts of drones discussed above would seem likely to create hostility towards conservation among the local population, which could undermine conservation effectiveness.

Hostility towards drones could result in direct actions being taken against them, for example by shooting them down. Such incidents have already been reported several times in the US, usually in cases where hunters shoot down drones that were deployed by animal welfare groups opposed to their actions (Schroyer 2014; Times and Democrat 2014). Similarly, a drone deployed by a German TV station to monitor illegal hunting in Malta was shot down in 2012 (CABS 2014; Times of Malta 2014). Demonstrating perhaps an emerging public discourse about drones and privacy violations, a recent US advertising campaign for the ‘Salvo 12’ shotgun silencer made by SilencerCo featured “Johnny Dronehunter” the “Defender of Privacy” shooting down drones with a shotgun in the desert (SilencerCo 2014). Investing money in drones only to have them shot down would be a negative outcome from a conservation perspective.

Hostility towards drones could also undermine the broader conditions for conservation success. The last twenty or so years of conservation practice have been characterised by efforts to move away from the so-called ‘fortress conservation’ or ‘fences and fines’ strategies based on exclusion and negative incentives towards more inclusive approaches that involve local people in conservation and share benefits with them^1^ (Adams 2004). These approaches, described *inter alia* as community conservation and community-based natural resource management, are built on the assumption that long-term conservation success requires support from local people rather than opposition (Hulme and Murphree 2001; Adams et al. 2004; Maffey et al. 2015). Drones might undermine this conservation paradigm by creating the impression, intended or otherwise, of a return to a militarised fortress conservation approach. It seems plausible, and even probable, that such perceptions of drones would make other conservation activities more difficult (Humle et al. 2014; Smith 2014). If such a situation came to pass, it might be very difficult to resolve, because rebuilding positive relationships with angered stakeholders can take a very long time, as is well established in the literature on human-wildlife conflict (Dickman 2010).

Whether or not the social risks associated with drones are recognised and taken seriously by conservation practitioners seems likely to depend on how the ‘success’ of conservation drones is framed (cf. Verma et al. 2015). If they are framed as a technical solution to a short-term technical problem (e.g. how to monitor illegal deforestation), they may well be ‘better’ than the alternatives because they are cheaper, more flexible, more efficient, etc. However, if drones are framed in a more holistic sense that takes into account social and political implications and a longer time frame, they may no longer be the best option, for some of the reasons identified above. A useful example here from outside conservation is the experience of efforts to introduce genetically modified (GM) crops into Europe. Seen by their developers as a brilliant technical solution to a set of technical problems around crop yields and pest resistance, GM crops were hailed as the answer to various agricultural crises. However, fears about their health and ecological risks led to widespread social concerns about GM crops, eventually resulting in them being banned within the EU (reviewed by Frewer et al. 2013). Rightly or wrongly, this has held back the introduction of GM crops for at least a decade in Europe, a situation that might perhaps have been avoided if their promoters had been more sensitive to how they might be perceived by important stakeholders. It seems quite plausible that a similar story may come to pass for conservation drones in some places if they are implemented without proper forethought.

## What can be done to minimise the social risks of conservation drones?

The conservation movement has made great efforts in recent years to recognise that its actions can cause harm to people and to minimise that harm in practice. This is exemplified by the framework declaration of the Conservation Initiative on Human Rights that has been signed by many leading international conservation charities. Some of the possible negative social impacts of drones identified above have the potential to infringe such agreements and undermine the good intentions of many conservation organisations and researchers. It might therefore be reasonable to expect that the conservation community should be thinking seriously about the possible harm that drones could do to people.

In fact, based on the literature I have reviewed for this paper, it is remarkable how little attention the conservation community has given to these issues. Only two papers (Paneque-Galvez et al. 2014; Humle et al. 2014) make any mention of possible negative ethical or privacy impacts of conservation drones. Perhaps tellingly, the only other paper to make a particular point about social impacts is concerned with how drones might be perceived by ecotourists, rather than by local people (Mulero-Pazmany et al. 2014). This analysis supports Duffy’s (2014) argument that “these technologies have been rolled out without really thinking through their wider social implications”. This may be a consequence of a sense that conservation is a force for good and therefore does not raise ethical issues, a lack of concern for marginalised publics in the global south relative to those in the global north, or perhaps the predominance of technical specialists developing and writing about drones who may not be familiar with social issues.

If conservation is to take action to mitigate the social risks of using drones, what could be done? The most obvious strategy would be to avoid using drones in places where there are people. However, this will rarely be possible, and even where there are no people there may be resources that people care about, meaning that the use of drones could still generate social impacts. Instead, I recommend as next steps a programme of new empirical research and the development of a framework for self-regulation of drone use by the conservation community.

### Research

At present, there is an almost complete lack of field data to confirm or disprove the possible social impacts of drones identified by this paper. There is therefore a clear need for research to fill this knowledge gap (as is the case for ‘digital conservation’ more broadly; Arts et al. 2015). Ideally, this should be empirical fieldwork that takes place at sites where drones are being implemented. Among other questions, such research could investigate: the social impacts of drones (e.g. are they welcomed, or do they cause alarm, fear or confusion? Are certain groups particularly affected? Do these impacts fade over time?); the relationship between the social context on the ground and social impacts of drones (e.g. population size, existing conflict); whether the design of drones or how they are communicated to different stakeholders affects such impacts; whether drones are more easily accepted under particular social conditions (e.g. participation of local people or where conservation actors are widely trusted and supported); and how the use of drones interacts with other conservation strategies (e.g. does using drones undermine efforts to improve relations with park neighbours through alternative livelihoods projects?). With the cooperation of those implementing drones, it may be possible to adopt an experimental research design in some cases, for example by altering the design of drones or how they are communicated in different areas. The results of such research would hopefully provide useful evidence on whether and under what circumstances drones are likely to have positive or negative social impacts. This information would be tremendously useful to practitioners.

### Regulation

The material reviewed in this paper demonstrates that the use of drones for conservation comes with the risk of negative social impacts. It therefore makes sense for their use to be regulated. Regulating drones is clearly challenging, because they are so variable in size and capabilities that it is “difficult to establish over-arching regulatory mechanisms” (Finn and Wright 2012, p. 186). Regulations governing drones are also inconsistent between countries and changing rapidly within countries. Given the confusing and rapidly shifting legal regulation of the use of drones, I recommend that the conservation sector should adopt a policy of self-regulation, at least until the legal status of drones becomes clearer. Self-regulation has been recommended in other cases of rapidly emerging technologies with potential risks, such as gene drives that seek to alter the genetic makeup of populations by adding, disrupting or editing genes (Oye et al. 2014). One advantage of effective self-regulation is that it may encourage states to avoid excessive over-regulation or complete bans, which have been criticised by advocates of drones on the grounds that preventing their use is allowing illegal activities that they might have prevented to continue (Koebler 2014). For example, writing about the situation in Kenya, Andrews (2014) states: “Thanks to the ban it’s now more negotiations and no doubt a horrible death for a few more rhinos.”

Self-regulation could be used to limit the use of drones to certain applications and under certain circumstances. For example, the use of drones for research purposes involving people might be required to go through the Free Prior and Informed Consent (FPIC) process, which is designed to ensure that before providing research data, human respondents are fully aware of the research and its aims, and have given their consent freely. The use of drones in areas containing human populations with very little prior contact with electronic technology might be limited to minimise cultural impacts (Paneque-Galvez et al. 2014). Projects using drones might be required to carry out a ‘privacy impact assessment’ in advance (Andrews 2014), and then to collect social monitoring data during implementation to ensure that any negative impacts are identified early and appropriate action taken. The use of drones for law enforcement purposes might be considered appropriate only for state law enforcement agencies and forbidden for private or NGO devices. Potentially sensitive images that are incidental to the purpose of the drone (e.g. people accidentally caught on camera) could be blurred or deleted before being released to other actors, a process that happens as standard with certain images on Google Streetview (e.g. all car number plates are blurred out before publication). Finally, regulations could encourage avoiding the use of the word ‘drone’ at all, given its militaristic associations.

Determining appropriate self-regulation of drone use by the conservation sector requires a process involving relevant experts from within and outside conservation. An appropriate vehicle for such a process might be the existing Conservation Initiative on Human Rights (CIHR), which involves many of the World’s largest conservation charities. Any resulting regulatory framework could perhaps be added as a new section to the CIHR. As Finn and Wright (2012, p. 194) argue, a key focus of deliberations around regulation should be the question of what drones “should do, rather than what they may do”.

## Conclusion

The use of drones for conservation has great potential to deliver conservation benefits. However, very little attention has been given to the social implications of their use. The material reviewed here suggests that they may have positive social impacts under certain circumstances, perhaps in particular where they are used by local people (Paneque-Galvez et al. 2014). However, drones have the potential to generate a range of negative social impacts. These impacts could contravene the stated intention of much of the conservation movement to abide by good ethical practice, and could undermine conservation effectiveness in the long term. It is therefore essential that new research be carried out to establish the real social impacts of drones and how problems could be mitigated, and for the conservation sector to agree on a sensible framework for the self-regulation of drone use. Until such research and regulation is in place, it would be wise to avoid rolling out the use of drones for conservation on a large scale.

Conservation has a rich history of seizing on new ideas as the solution to all its problems (Redford et al. 2013). In many, if not most, cases, the latest fad has turned out to be less effective than was hoped (Redford et al. 2013). For example, electric fences, another widely supported technological intervention for conservation, have created long-term antagonism with local communities in Namibia to the detriment of conservation objectives (Hoole and Berkes 2010). It may be tempting to think of conservation drones as a ‘silver bullet’ solution to problems of monitoring and enforcement, but earlier experience and the possible social implications reviewed in this paper suggest that they should be used with caution. They are likely to work well in some places, but in others they may make things worse. Even where they are effective, they may distract attention from the social and political realities (and conflicts) in which conservation problems exist. Ultimately, they are unlikely to replace the ongoing need for tried and tested conservation strategies to address these problems on the ground, such as community meetings, shared decision making and efforts to understand the root causes of conservation problems (Humle et al. 2014).

## Footnotes

1 Although some have argued for a return to fortress conservation (e.g. Terborgh 1999) and others that it never went away (e.g. Duffy 2014).
